# Special Issue “Synthesis and Applications of Oligonucleotide Conjugates”

**DOI:** 10.3390/molecules22101700

**Published:** 2017-10-13

**Authors:** Harri Lönnberg

**Affiliations:** Department of Chemistry, University of Turku, FIN-20014 Turku, Finland; harlon@utu.fi

The underlying idea of oligonucleotide conjugates is to provide oligonucleotide with some novel property. This is achieved by tethering an unnatural organic structure and a naturally occurring small molecule of a biopolymer fragment to an unmodified or structurally modified oligonucleotide. The growing interest in oligonucleotide-based drugs during the past two decades has increased the interest in utilization of their covalently conjugated derivatives. In line with this, all the papers in this special issue have a link to either drug development or diagnostics.

The role of the oligonucleotide moiety in their conjugate is to ensure specific binding to a desired biological target. This is usually based on recognition of a single-stranded nucleic acid target by Watson-Crick base-pairing, and a double-stranded target by Hoogsteen or combined Hoogsteen and Watson Crick pairing. Oligonucleotides may, however, also be used to recognize proteins or even structures of cell surfaces. This is based on preparation of the so-called aptamers by chemical evolution. Aptamers have a protein-like 3D structure, and very high affinity to the agent used as a target in the selection process from a random pool of oligonucleotide sequences. The conjugate group may, in turn, be aimed at allowing sensitive detection in vivo, site-selective cleavage or cross-linking with the target, cell type recognition, better cellular uptake, control of intracellular localization or enhanced release from endosomes, just to mention a few examples. Applications of conjugates based on both the classical double helix formation and aptamer binding are discussed in the present special issue.

The design, preparation and applications of aptamer conjugates are reviewed in the present special issue [1]. Their synthesis by derivatization of aptamer functionalities with reporter groups, nanoparticles, chemotherapeutic agents and oligonucleotides, as well as the applications of these multivalent constructs as biosensors, versatile research tools for cell biology and candidates of oligonucleotide-based therapeutics have been discussed. In addition, two novel diagnostic applications based on aptamers are introduced. A fluorescent biosensor platform based on sequential use of two different aptamers for detection of Shigella sonnei pathogen has been developed [2]. An amino-functionalized aptamer selected against this pathogen is immobilized to a solid phase and used to selectively collect Shigella sonnei cells. The bound bacteria are then visualized with another fluorescently labelled aptamer. The method has been shown to be able to discriminate Shigella sonnei from other Shigella genus species and intestinal bacteria. Another paper [3] concerns development of a DNA type aptamer recognizing Gremlin-1, a phosphorylated glycoprotein playing a vital role in several cellular processes. The aptamer is sufficiently stable under cell culture conditions to allow application of conventional techniques of cell biology and detection of subcellular location of Gremlin-1. New oligonucleotide probes developed for non-denaturing fluorescence in situ hybridization detection of wheat chromosomes offer a novel application of conventional hybridization diagnostics [4]. The probes enable identification of Dasypyrum villosum, a species of annual grass, from wheat chromosomes, and to distinguish individual D. villosum chromosomes.

Chimeric RNA molecules consisting of an aptamer domain and a hammerhead ribozyme domain have been introduced as potent candidates for development of novel type drugs against RNA viruses [5]. The aptamer domain was evolved by in vitro selection for binding a conserved domain of HCV genome and simultaneously allowing cleavage by the hammerhead domain. The chimeras obtained inhibited viral translation and replication in cell culture, serving as a proof of concept for further development towards more stable inhibitory RNA by introduction of structurally modified building blocks. Another interesting report on therapeutic oligonucleotides deals with preparation and cell culture studies of high metallacarborane-loaded antisense oligonucleotides useful in cancer boron neutron capture therapy (BNCT) [6]. The conjugates were obtained by subjecting a prefabricated oligonucleotide bearing several 2′-*O*-(pro-2-yn-1-yl) groups to Cu(I) catalyzed click reaction with azido functionalized boron clusters, [(3,3′-iron-1,2,1′,2′-dicarbollide)(−1)]ates]. When targeted to epidermal growth factor receptor, the conjugate retained the silencing capacity of the unmodified oligonucleotide and exhibited anti-oxidant activity. Accordingly, the conjugates show promise for development of therapeutic agents exhibiting combined antisense/anti-oxidant and BNCT activities. The third paper on therapeutic oligonucleotides describes an integrin specific transfection agent for delivery of siRNA to tumor cells [7]. The agent was a chimeric peptide containing two cyclic structures that incorporated an Arg-Gly-Asp motif for integrin recognition and a cell penetrating arginine nonamer for enhancement of cellular uptake. This peptide was shown to internalize siRNA into HepG2 cells in vitro and to result in gene silencing.

The rest of the papers in this special issue deal with synthetic methods of oligonucleotide conjugates. Cu(I) promoted click chemistry has been used to derivatize oligonucleotides incorporating 2′-*N*-alkyne-2′-amino-LNA nucleosides with azido functionalized galactose or triantennary galactose cluster [8]. The conjugates obtained showed enhanced affinity and good selectivity towards complementary DNA/RNA strands. A phosphoramidite building block of ribostamycin that allow incorporation of this aminoglycoside-aminocyclitol antibiotic in any position of oligonucleotide has been synthesized [9]. The intrachain 2′-*O*-methyl oligoribonucleotide conjugate obtained has been shown to for a stable clamp-type triplex with DNA. Finally, preparation of triantennary N-acetylgalactosmine conjugates of antisense oligonucleotide conjugate in solution phase and on a solid support has been compared [10]. This study receives its importance from the use of this conjugate group for delivery of therapeutic oligonucleotides to liver.
